# (*E*)-1-[(Diphenyl­amino)meth­yl]-4-(4-fluoro­benzyl­ideneamino)-3-[1-(4-iso­butyl­phen­yl)eth­yl]-1*H*-1,2,4-triazole-5(4*H*)-thione

**DOI:** 10.1107/S1600536809052039

**Published:** 2009-12-09

**Authors:** Jia Hao Goh, Hoong-Kun Fun, A. C. Vinayaka, B. Kalluraya

**Affiliations:** a X-ray Crystallography Unit, School of Physics, Universiti Sains Malaysia, 11800 USM, Penang, Malaysia; b Department of Studies in Chemistry, Mangalore University, Mangalagangotri, Mangalore 574 199, India

## Abstract

The title 1,2,4-triazole compound, C_34_H_34_FN_5_S, exists in a *trans* configuration with respect to the acyclic C=N bond. An intra­molecular C—H⋯S contact generates a six-membered ring, producing an *S*(6) ring motif. The essentially planar 1,2,4-triazole ring [maximum deviation 0.008 (1) Å] is inclined at 21.43 (5) and 83.03 (6)°, respectively, with respect to the flurophenyl unit and the isobutyl-substituted benzene ring. The diphenyl­amino unit is not planar, as indicated by the dihedral angle between two phenyl rings of 76.95 (6)°. The crystal structure is stabilized by C—H⋯π and π–π [centroid–centroid distance = 3.6169 (6) Å] inter­actions; mol­ecules are stacked along the *b* axis.

## Related literature

For general background to and applications of 1,2,4-triazole derivatives, see: Calhoun *et al.* (1995 ▶); Pandeya *et al.* (1999 ▶, 2000 ▶); Sujith *et al.* (2009 ▶). For hydrogen-bond motifs, see: Bernstein *et al.* (1995 ▶). For a closely related structure, see: Goh *et al.* (2010 ▶). For the stability of the temperature controller used for the data collection, see: Cosier & Glazer (1986 ▶).
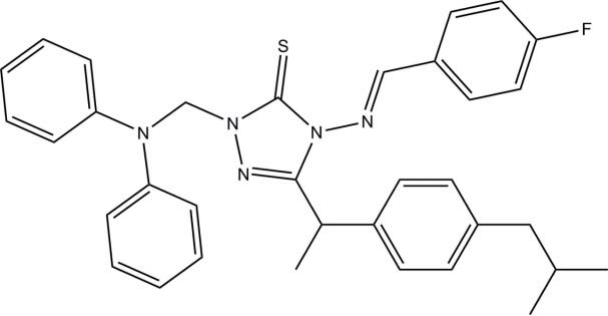


## Experimental

### 

#### Crystal data

C_34_H_34_FN_5_S
*M* *_r_* = 563.72
Monoclinic, 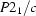

*a* = 10.8175 (2) Å
*b* = 9.9579 (1) Å
*c* = 27.8344 (4) Å
β = 105.199 (1)°
*V* = 2893.43 (7) Å^3^
*Z* = 4
Mo *K*α radiation
μ = 0.15 mm^−1^
*T* = 100 K
0.50 × 0.36 × 0.32 mm

#### Data collection

Bruker SMART APEXII CCD area-detector diffractometer
Absorption correction: multi-scan (*SADABS*; Bruker, 2005 ▶) *T*_min_ = 0.928, *T*_max_ = 0.953
58342 measured reflections
10414 independent reflections
8240 reflections with *I* > 2σ(*I*)
*R*_int_ = 0.035

#### Refinement

*R*[*F* ^2^ > 2σ(*F* ^2^)] = 0.044
*wR*(*F* ^2^) = 0.120
*S* = 1.04
10414 reflections
373 parameters
H-atom parameters constrained
Δρ_max_ = 0.42 e Å^−3^
Δρ_min_ = −0.25 e Å^−3^

### 

Data collection: *APEX2* (Bruker, 2005 ▶); cell refinement: *SAINT* (Bruker, 2005 ▶); data reduction: *SAINT*; program(s) used to solve structure: *SHELXTL* (Sheldrick, 2008 ▶); program(s) used to refine structure: *SHELXTL*; molecular graphics: *SHELXTL*; software used to prepare material for publication: *SHELXTL* and *PLATON* (Spek, 2009 ▶).

## Supplementary Material

Crystal structure: contains datablocks global, I. DOI: 10.1107/S1600536809052039/tk2593sup1.cif

Structure factors: contains datablocks I. DOI: 10.1107/S1600536809052039/tk2593Isup2.hkl

Additional supplementary materials:  crystallographic information; 3D view; checkCIF report

## Figures and Tables

**Table 1:** Hydrogen-bond geometry (Å, °)

*D*—H⋯*A*	*D*—H	H⋯*A*	*D*⋯*A*	*D*—H⋯*A*
C7—H7*A*⋯S1	0.93	2.51	3.1983 (11)	131
C4—H4*A*⋯*Cg*1^i^	0.93	2.65	3.5688 (13)	169
C20—H20*C*⋯*Cg*2^ii^	0.96	2.99	3.9291 (12)	166

Symmetry codes: (i) 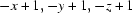; (ii) 
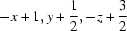
. *Cg*1 and *Cg*2 are the centroids of the C11–C16 and C29–C34 benzene rings, respectively.

**Fig. 1.**
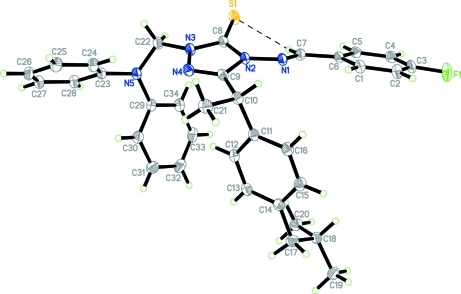
The molecular structure of the title compound, showing 50% probability displacement ellipsoids for non-H atoms and the atom-numbering scheme. An intramolecular contact is shown as dashed line.

**Fig. 2.**
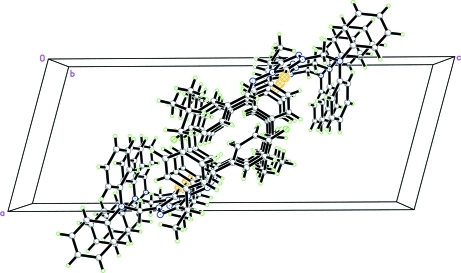
Part of the crystal structure of the title compound, viewed along the b axis, showing molecules being stacked along the b axis.

## supplementary crystallographic information

### Comment

Non-steroidal anti-inflammatory drugs (NSAIDs) are widely used. Despite their large therapeutic applications, they have several undesired, often serious side-effects (Calhoun *et al.*, 1995) so long-term administration is not advisable. Thus, the need for new anti-inflammatory drugs is obvious and accordingly, there has been renewed interest in anti-inflammatory agents endowed with potent biological activity. In this context, it has been shown that some Mannich bases find applications as anti-inflammatory analgesic agents (Sujith *et al.*, 2009) and anti-microbial activities (Pandeya *et al.*, 1999, 2000).

The title 1,2,4-triazole derivative (Fig. 1) exists in an *E* configuration with respect to the acyclic C7═N1 bond [bond length of C7═N1 = 1.2824 (14) Å and torsion angle of C6–C7–N1–N2 = -177.20 (9)°]. An intramolecular C7—H7A···S1 contact generates a six-membered ring, producing an *S*(6) ring motif (Bernstein *et al.*, 1995). The 1,2,4-triazole ring (N2/C8/N3/N4/C9) is essentially planar, with maximum deviation of 0.008 (1) for atom N2. The 1,2,4-triazole ring is inclined at dihedral angles of 21.43 (5) and 83.03 (6)°, respectively, to the fluorophenyl moiety (C1-C6/F1) and isobutyl-substituted phenyl ring (C11-C16). The diphenylamino moiety is not planar, as indicated by the dihedral angle formed between two phenyl rings (C23-C28 and C29-C34) of 76.95 (6)°. The bond lengths and angles are within normal ranges and comparable to a closely related structure (Goh *et al.*, 2010).

In the crystal structure, molecules are stacked along the *b* axis (Fig. 2). Intermolecular C4—H4A···*Cg*1, C20—H20C···*Cg*2 (Table 1) as well as *Cg*3···*Cg*3 interactions stabilize the crystal structure [*Cg*3···*Cg*3 = 3.6169 (6)^i^; (i) 1-x, 1-y, 1-z; *Cg*1, *Cg*2 and *Cg*3 are the centroids of C11-C16, C29-C34 and C1-C6 phenyl rings, respectively].

### Experimental

The title 1,2,4-triazole compound was obtained by the Mannich reaction of Schiff base (0.01 mol), formaldehyde (40 %, 1 ml) and *N*,*N*-diphenylamine (0.01 mol) in ethanol (10 ml) after stirring at room temperature for 20 h. The solid product obtained was collected by filtration, washed with ethanol and dried. Colourless single crystals were obtained from a 1:2 mixture of *N,N*-dimethylformamide and ethanol by slow evaporation.

### Refinement

All hydrogen atoms were placed in their calculated positions, with C—H = 0.93 – 0.97 Å, and refined using a riding model with *U*_iso_ = 1.2 or 1.5 *U*_eq_(C). A rotating group model was used for the methyl groups.

### Crystal data

**Table:**

C_34_H_34_FN_5_S	*F*(000) = 1192
*M**_r_* = 563.72	*D*_x_ = 1.294 Mg m^−^^3^
Monoclinic, *P*2_1_/*c*	Mo *K*α radiation, λ = 0.71073 Å
Hall symbol: -P 2ybc	Cell parameters from 9739 reflections
*a* = 10.8175 (2) Å	θ = 2.6–34.7°
*b* = 9.9579 (1) Å	µ = 0.15 mm^−^^1^
*c* = 27.8344 (4) Å	*T* = 100 K
β = 105.199 (1)°	Block, colourless
*V* = 2893.43 (7) Å^3^	0.50 × 0.36 × 0.32 mm
*Z* = 4	

### Data collection

**Table:**

Bruker SMART APEXII CCD area-detector diffractometer	10414 independent reflections
Radiation source: fine-focus sealed tube	8240 reflections with *I* > 2σ(*I*)
graphite	*R*_int_ = 0.035
φ and ω scans	θ_max_ = 32.5°, θ_min_ = 2.1°
Absorption correction: multi-scan (*SADABS*; Bruker, 2005)	*h* = −16→16
*T*_min_ = 0.928, *T*_max_ = 0.953	*k* = −15→14
58342 measured reflections	*l* = −42→41

### Refinement

**Table:**

Refinement on *F*^2^	Primary atom site location: structure-invariant direct methods
Least-squares matrix: full	Secondary atom site location: difference Fourier map
*R*[*F*^2^ > 2σ(*F*^2^)] = 0.044	Hydrogen site location: inferred from neighbouring sites
*wR*(*F*^2^) = 0.120	H-atom parameters constrained
*S* = 1.04	*w* = 1/[σ^2^(*F*_o_^2^) + (0.0575*P*)^2^ + 0.8906*P*] where *P* = (*F*_o_^2^ + 2*F*_c_^2^)/3
10414 reflections	(Δ/σ)_max_ < 0.001
373 parameters	Δρ_max_ = 0.42 e Å^−^^3^
0 restraints	Δρ_min_ = −0.25 e Å^−^^3^

### Special details

**Table:**

Experimental. The crystal was placed in the cold stream of an Oxford Cyrosystems Cobra open-flow nitrogen cryostat (Cosier & Glazer, 1986) operating at 100.0 (1)K.
Geometry. All esds (except the esd in the dihedral angle between two l.s. planes) are estimated using the full covariance matrix. The cell esds are taken into account individually in the estimation of esds in distances, angles and torsion angles; correlations between esds in cell parameters are only used when they are defined by crystal symmetry. An approximate (isotropic) treatment of cell esds is used for estimating esds involving l.s. planes.
Refinement. Refinement of F^2^ against ALL reflections. The weighted R-factor wR and goodness of fit S are based on F^2^, conventional R-factors R are based on F, with F set to zero for negative F^2^. The threshold expression of F^2^ > 2sigma(F^2^) is used only for calculating R-factors(gt) etc. and is not relevant to the choice of reflections for refinement. R-factors based on F^2^ are statistically about twice as large as those based on F, and R- factors based on ALL data will be even larger.

### Fractional atomic coordinates and isotropic or equivalent isotropic displacement parameters (Å^2^)

**Table:**

	*x*	*y*	*z*	*U*_iso_*/*U*_eq_	
S1	0.16117 (3)	0.22751 (3)	0.589154 (11)	0.02485 (7)	
F1	0.52953 (8)	0.48389 (9)	0.37293 (3)	0.03681 (19)	
N1	0.17190 (9)	0.50769 (9)	0.51987 (3)	0.01794 (17)	
N2	0.11972 (9)	0.49448 (9)	0.56030 (3)	0.01672 (16)	
N3	0.04413 (9)	0.43808 (9)	0.62106 (3)	0.01820 (17)	
N4	0.01010 (9)	0.57105 (9)	0.61131 (3)	0.01838 (17)	
N5	0.05479 (9)	0.45860 (10)	0.70862 (3)	0.02075 (18)	
C1	0.29376 (11)	0.54223 (11)	0.44134 (4)	0.0207 (2)	
H1A	0.2273	0.6013	0.4416	0.025*	
C2	0.36276 (12)	0.55679 (12)	0.40618 (4)	0.0244 (2)	
H2A	0.3438	0.6255	0.3828	0.029*	
C3	0.46049 (12)	0.46680 (12)	0.40667 (4)	0.0242 (2)	
C4	0.49201 (11)	0.36250 (11)	0.43997 (4)	0.0227 (2)	
H4A	0.5576	0.3030	0.4390	0.027*	
C5	0.42230 (11)	0.34904 (11)	0.47520 (4)	0.0200 (2)	
H5A	0.4415	0.2793	0.4982	0.024*	
C6	0.32382 (10)	0.43867 (10)	0.47658 (4)	0.01744 (18)	
C7	0.26117 (10)	0.42571 (10)	0.51707 (4)	0.01848 (19)	
H7A	0.2861	0.3581	0.5407	0.022*	
C8	0.11087 (10)	0.38574 (10)	0.59048 (4)	0.01786 (19)	
C9	0.05599 (10)	0.60207 (10)	0.57402 (4)	0.01684 (18)	
C10	0.04846 (10)	0.73715 (10)	0.55006 (4)	0.01775 (19)	
H10A	0.0237	0.7246	0.5139	0.021*	
C11	0.17695 (10)	0.80893 (10)	0.56429 (4)	0.01719 (18)	
C12	0.24975 (11)	0.81605 (11)	0.61379 (4)	0.0201 (2)	
H12A	0.2251	0.7669	0.6381	0.024*	
C13	0.35875 (11)	0.89604 (11)	0.62690 (4)	0.0211 (2)	
H13A	0.4056	0.9002	0.6601	0.025*	
C14	0.39935 (11)	0.97024 (10)	0.59134 (4)	0.0202 (2)	
C15	0.32793 (12)	0.95927 (11)	0.54167 (4)	0.0221 (2)	
H15A	0.3543	1.0058	0.5171	0.026*	
C16	0.21845 (11)	0.88019 (11)	0.52838 (4)	0.0204 (2)	
H16A	0.1724	0.8748	0.4952	0.024*	
C17	0.51678 (11)	1.05862 (11)	0.60507 (4)	0.0229 (2)	
H17A	0.5272	1.0919	0.6386	0.028*	
H17B	0.5030	1.1355	0.5829	0.028*	
C18	0.64135 (11)	0.98818 (10)	0.60244 (4)	0.0200 (2)	
H18A	0.6264	0.9450	0.5698	0.024*	
C19	0.74893 (12)	1.09067 (12)	0.60727 (5)	0.0260 (2)	
H19A	0.8259	1.0454	0.6054	0.039*	
H19B	0.7634	1.1361	0.6387	0.039*	
H19C	0.7251	1.1549	0.5807	0.039*	
C20	0.68007 (12)	0.87972 (12)	0.64228 (4)	0.0241 (2)	
H20A	0.7545	0.8334	0.6382	0.036*	
H20B	0.6109	0.8170	0.6390	0.036*	
H20C	0.6991	0.9205	0.6746	0.036*	
C21	−0.05414 (11)	0.82343 (11)	0.56409 (5)	0.0238 (2)	
H21A	−0.1359	0.7798	0.5531	0.036*	
H21B	−0.0580	0.9097	0.5484	0.036*	
H21C	−0.0328	0.8348	0.5996	0.036*	
C22	0.02287 (12)	0.37238 (11)	0.66559 (4)	0.0217 (2)	
H22A	−0.0663	0.3461	0.6590	0.026*	
H22B	0.0746	0.2916	0.6726	0.026*	
C23	−0.04449 (10)	0.50037 (10)	0.73041 (4)	0.01839 (19)	
C24	−0.15516 (11)	0.56251 (12)	0.70258 (4)	0.0222 (2)	
H24A	−0.1661	0.5789	0.6688	0.027*	
C25	−0.24961 (11)	0.60002 (12)	0.72568 (4)	0.0240 (2)	
H25A	−0.3246	0.6400	0.7070	0.029*	
C26	−0.23290 (11)	0.57828 (11)	0.77618 (4)	0.0218 (2)	
H26A	−0.2961	0.6044	0.7914	0.026*	
C27	−0.12167 (11)	0.51754 (11)	0.80400 (4)	0.0218 (2)	
H27A	−0.1100	0.5035	0.8379	0.026*	
C28	−0.02782 (11)	0.47779 (11)	0.78124 (4)	0.0209 (2)	
H28A	0.0462	0.4361	0.7998	0.025*	
C29	0.17503 (10)	0.52279 (11)	0.72258 (4)	0.0194 (2)	
C30	0.18674 (11)	0.64932 (11)	0.74534 (4)	0.0215 (2)	
H30A	0.1152	0.6899	0.7516	0.026*	
C31	0.30399 (12)	0.71451 (12)	0.75856 (5)	0.0258 (2)	
H31A	0.3105	0.7984	0.7737	0.031*	
C32	0.41226 (12)	0.65524 (13)	0.74936 (5)	0.0277 (2)	
H32A	0.4910	0.6988	0.7583	0.033*	
C33	0.40086 (12)	0.53055 (13)	0.72666 (5)	0.0267 (2)	
H33A	0.4729	0.4904	0.7206	0.032*	
C34	0.28381 (11)	0.46417 (12)	0.71282 (4)	0.0235 (2)	
H34A	0.2777	0.3811	0.6971	0.028*	

### Atomic displacement parameters (Å^2^)

**Table:**

	*U*^11^	*U*^22^	*U*^33^	*U*^12^	*U*^13^	*U*^23^
S1	0.03825 (17)	0.01658 (12)	0.02449 (14)	0.00605 (11)	0.01669 (12)	0.00197 (9)
F1	0.0399 (5)	0.0505 (5)	0.0279 (4)	0.0028 (4)	0.0231 (3)	−0.0001 (3)
N1	0.0216 (4)	0.0189 (4)	0.0153 (4)	0.0013 (3)	0.0085 (3)	−0.0008 (3)
N2	0.0204 (4)	0.0164 (4)	0.0151 (4)	0.0031 (3)	0.0079 (3)	0.0002 (3)
N3	0.0224 (4)	0.0173 (4)	0.0167 (4)	0.0026 (3)	0.0083 (3)	0.0001 (3)
N4	0.0202 (4)	0.0174 (4)	0.0183 (4)	0.0030 (3)	0.0063 (3)	−0.0005 (3)
N5	0.0199 (4)	0.0249 (4)	0.0198 (4)	−0.0003 (3)	0.0093 (3)	−0.0036 (3)
C1	0.0223 (5)	0.0227 (5)	0.0179 (5)	0.0033 (4)	0.0067 (4)	0.0004 (4)
C2	0.0275 (6)	0.0289 (5)	0.0177 (5)	0.0015 (5)	0.0075 (4)	0.0018 (4)
C3	0.0256 (5)	0.0318 (6)	0.0185 (5)	−0.0022 (5)	0.0117 (4)	−0.0060 (4)
C4	0.0221 (5)	0.0228 (5)	0.0253 (5)	0.0008 (4)	0.0097 (4)	−0.0072 (4)
C5	0.0210 (5)	0.0177 (4)	0.0222 (5)	0.0008 (4)	0.0073 (4)	−0.0030 (4)
C6	0.0181 (5)	0.0181 (4)	0.0167 (4)	−0.0001 (4)	0.0057 (4)	−0.0028 (3)
C7	0.0193 (5)	0.0192 (4)	0.0180 (5)	0.0005 (4)	0.0067 (4)	−0.0001 (3)
C8	0.0216 (5)	0.0174 (4)	0.0157 (4)	0.0016 (4)	0.0069 (4)	−0.0007 (3)
C9	0.0169 (4)	0.0173 (4)	0.0164 (4)	0.0024 (4)	0.0044 (4)	−0.0022 (3)
C10	0.0200 (5)	0.0168 (4)	0.0165 (4)	0.0041 (4)	0.0048 (4)	0.0006 (3)
C11	0.0196 (5)	0.0155 (4)	0.0176 (4)	0.0043 (4)	0.0067 (4)	0.0005 (3)
C12	0.0222 (5)	0.0230 (5)	0.0166 (5)	0.0018 (4)	0.0078 (4)	0.0003 (4)
C13	0.0223 (5)	0.0248 (5)	0.0167 (5)	0.0009 (4)	0.0061 (4)	−0.0021 (4)
C14	0.0217 (5)	0.0168 (4)	0.0230 (5)	0.0024 (4)	0.0076 (4)	−0.0005 (4)
C15	0.0268 (5)	0.0195 (4)	0.0209 (5)	0.0020 (4)	0.0082 (4)	0.0046 (4)
C16	0.0248 (5)	0.0196 (4)	0.0165 (4)	0.0032 (4)	0.0050 (4)	0.0032 (3)
C17	0.0244 (5)	0.0181 (4)	0.0267 (5)	−0.0001 (4)	0.0072 (4)	−0.0016 (4)
C18	0.0246 (5)	0.0188 (4)	0.0180 (5)	0.0005 (4)	0.0078 (4)	0.0000 (4)
C19	0.0278 (6)	0.0225 (5)	0.0312 (6)	−0.0012 (4)	0.0139 (5)	0.0010 (4)
C20	0.0252 (5)	0.0221 (5)	0.0234 (5)	−0.0010 (4)	0.0036 (4)	0.0024 (4)
C21	0.0211 (5)	0.0207 (5)	0.0294 (6)	0.0055 (4)	0.0064 (4)	−0.0008 (4)
C22	0.0275 (5)	0.0204 (5)	0.0198 (5)	−0.0007 (4)	0.0111 (4)	−0.0004 (4)
C23	0.0191 (5)	0.0188 (4)	0.0193 (5)	−0.0005 (4)	0.0086 (4)	−0.0014 (3)
C24	0.0248 (5)	0.0246 (5)	0.0185 (5)	0.0014 (4)	0.0078 (4)	0.0024 (4)
C25	0.0224 (5)	0.0253 (5)	0.0249 (5)	0.0038 (4)	0.0074 (4)	0.0034 (4)
C26	0.0211 (5)	0.0224 (5)	0.0247 (5)	0.0006 (4)	0.0113 (4)	−0.0011 (4)
C27	0.0239 (5)	0.0247 (5)	0.0183 (5)	0.0007 (4)	0.0083 (4)	0.0001 (4)
C28	0.0201 (5)	0.0248 (5)	0.0182 (5)	0.0025 (4)	0.0058 (4)	0.0007 (4)
C29	0.0195 (5)	0.0218 (5)	0.0184 (5)	0.0018 (4)	0.0077 (4)	0.0024 (4)
C30	0.0213 (5)	0.0212 (5)	0.0234 (5)	0.0015 (4)	0.0086 (4)	0.0018 (4)
C31	0.0264 (6)	0.0245 (5)	0.0272 (6)	−0.0026 (4)	0.0079 (5)	0.0012 (4)
C32	0.0205 (5)	0.0358 (6)	0.0272 (6)	−0.0036 (5)	0.0069 (4)	0.0043 (5)
C33	0.0209 (5)	0.0369 (6)	0.0247 (5)	0.0046 (5)	0.0104 (4)	0.0050 (5)
C34	0.0235 (5)	0.0268 (5)	0.0223 (5)	0.0035 (4)	0.0100 (4)	0.0008 (4)

### Geometric parameters (Å, °)

**Table:**

S1—C8	1.6705 (11)	C17—C18	1.5378 (16)
F1—C3	1.3552 (13)	C17—H17A	0.9700
N1—C7	1.2824 (14)	C17—H17B	0.9700
N1—N2	1.3910 (12)	C18—C20	1.5257 (15)
N2—C9	1.3809 (13)	C18—C19	1.5271 (16)
N2—C8	1.3892 (13)	C18—H18A	0.9800
N3—C8	1.3562 (13)	C19—H19A	0.9600
N3—N4	1.3821 (12)	C19—H19B	0.9600
N3—C22	1.4720 (14)	C19—H19C	0.9600
N4—C9	1.2999 (14)	C20—H20A	0.9600
N5—C29	1.4095 (15)	C20—H20B	0.9600
N5—C23	1.4267 (14)	C20—H20C	0.9600
N5—C22	1.4401 (14)	C21—H21A	0.9600
C1—C2	1.3854 (16)	C21—H21B	0.9600
C1—C6	1.4014 (15)	C21—H21C	0.9600
C1—H1A	0.9300	C22—H22A	0.9700
C2—C3	1.3832 (17)	C22—H22B	0.9700
C2—H2A	0.9300	C23—C24	1.3891 (16)
C3—C4	1.3742 (17)	C23—C28	1.3968 (15)
C4—C5	1.3914 (16)	C24—C25	1.3932 (16)
C4—H4A	0.9300	C24—H24A	0.9300
C5—C6	1.3980 (15)	C25—C26	1.3863 (16)
C5—H5A	0.9300	C25—H25A	0.9300
C6—C7	1.4650 (15)	C26—C27	1.3874 (16)
C7—H7A	0.9300	C26—H26A	0.9300
C9—C10	1.4940 (14)	C27—C28	1.3879 (15)
C10—C11	1.5202 (15)	C27—H27A	0.9300
C10—C21	1.5330 (15)	C28—H28A	0.9300
C10—H10A	0.9800	C29—C30	1.4010 (16)
C11—C16	1.3926 (15)	C29—C34	1.4025 (15)
C11—C12	1.3983 (15)	C30—C31	1.3859 (17)
C12—C13	1.3900 (16)	C30—H30A	0.9300
C12—H12A	0.9300	C31—C32	1.3939 (18)
C13—C14	1.3953 (16)	C31—H31A	0.9300
C13—H13A	0.9300	C32—C33	1.3839 (19)
C14—C15	1.4000 (16)	C32—H32A	0.9300
C14—C17	1.5098 (16)	C33—C34	1.3902 (17)
C15—C16	1.3891 (16)	C33—H33A	0.9300
C15—H15A	0.9300	C34—H34A	0.9300
C16—H16A	0.9300		
			
C7—N1—N2	117.44 (9)	H17A—C17—H17B	107.6
C9—N2—C8	108.60 (9)	C20—C18—C19	110.58 (10)
C9—N2—N1	119.22 (8)	C20—C18—C17	111.43 (9)
C8—N2—N1	132.06 (8)	C19—C18—C17	110.37 (9)
C8—N3—N4	113.78 (9)	C20—C18—H18A	108.1
C8—N3—C22	125.73 (9)	C19—C18—H18A	108.1
N4—N3—C22	119.91 (8)	C17—C18—H18A	108.1
C9—N4—N3	104.27 (8)	C18—C19—H19A	109.5
C29—N5—C23	119.58 (9)	C18—C19—H19B	109.5
C29—N5—C22	120.16 (9)	H19A—C19—H19B	109.5
C23—N5—C22	118.96 (9)	C18—C19—H19C	109.5
C2—C1—C6	120.16 (10)	H19A—C19—H19C	109.5
C2—C1—H1A	119.9	H19B—C19—H19C	109.5
C6—C1—H1A	119.9	C18—C20—H20A	109.5
C3—C2—C1	118.50 (11)	C18—C20—H20B	109.5
C3—C2—H2A	120.7	H20A—C20—H20B	109.5
C1—C2—H2A	120.7	C18—C20—H20C	109.5
F1—C3—C4	118.56 (11)	H20A—C20—H20C	109.5
F1—C3—C2	118.06 (11)	H20B—C20—H20C	109.5
C4—C3—C2	123.37 (11)	C10—C21—H21A	109.5
C3—C4—C5	117.63 (10)	C10—C21—H21B	109.5
C3—C4—H4A	121.2	H21A—C21—H21B	109.5
C5—C4—H4A	121.2	C10—C21—H21C	109.5
C4—C5—C6	121.03 (10)	H21A—C21—H21C	109.5
C4—C5—H5A	119.5	H21B—C21—H21C	109.5
C6—C5—H5A	119.5	N5—C22—N3	112.27 (9)
C5—C6—C1	119.29 (10)	N5—C22—H22A	109.1
C5—C6—C7	118.32 (10)	N3—C22—H22A	109.1
C1—C6—C7	122.27 (10)	N5—C22—H22B	109.1
N1—C7—C6	119.89 (10)	N3—C22—H22B	109.1
N1—C7—H7A	120.1	H22A—C22—H22B	107.9
C6—C7—H7A	120.1	C24—C23—C28	120.00 (10)
N3—C8—N2	102.26 (8)	C24—C23—N5	121.85 (10)
N3—C8—S1	127.33 (8)	C28—C23—N5	118.16 (10)
N2—C8—S1	130.34 (8)	C23—C24—C25	119.42 (10)
N4—C9—N2	111.06 (9)	C23—C24—H24A	120.3
N4—C9—C10	125.65 (9)	C25—C24—H24A	120.3
N2—C9—C10	123.22 (9)	C26—C25—C24	120.64 (11)
C9—C10—C11	111.58 (8)	C26—C25—H25A	119.7
C9—C10—C21	110.44 (9)	C24—C25—H25A	119.7
C11—C10—C21	110.10 (9)	C25—C26—C27	119.83 (11)
C9—C10—H10A	108.2	C25—C26—H26A	120.1
C11—C10—H10A	108.2	C27—C26—H26A	120.1
C21—C10—H10A	108.2	C26—C27—C28	120.04 (10)
C16—C11—C12	118.50 (10)	C26—C27—H27A	120.0
C16—C11—C10	119.83 (9)	C28—C27—H27A	120.0
C12—C11—C10	121.39 (9)	C27—C28—C23	120.06 (10)
C13—C12—C11	120.51 (10)	C27—C28—H28A	120.0
C13—C12—H12A	119.7	C23—C28—H28A	120.0
C11—C12—H12A	119.7	C30—C29—C34	118.75 (10)
C12—C13—C14	121.35 (10)	C30—C29—N5	120.00 (10)
C12—C13—H13A	119.3	C34—C29—N5	121.22 (10)
C14—C13—H13A	119.3	C31—C30—C29	120.61 (11)
C13—C14—C15	117.67 (10)	C31—C30—H30A	119.7
C13—C14—C17	121.98 (10)	C29—C30—H30A	119.7
C15—C14—C17	120.35 (10)	C30—C31—C32	120.49 (11)
C16—C15—C14	121.23 (10)	C30—C31—H31A	119.8
C16—C15—H15A	119.4	C32—C31—H31A	119.8
C14—C15—H15A	119.4	C33—C32—C31	119.02 (11)
C15—C16—C11	120.69 (10)	C33—C32—H32A	120.5
C15—C16—H16A	119.7	C31—C32—H32A	120.5
C11—C16—H16A	119.7	C32—C33—C34	121.25 (11)
C14—C17—C18	114.31 (9)	C32—C33—H33A	119.4
C14—C17—H17A	108.7	C34—C33—H33A	119.4
C18—C17—H17A	108.7	C33—C34—C29	119.86 (11)
C14—C17—H17B	108.7	C33—C34—H34A	120.1
C18—C17—H17B	108.7	C29—C34—H34A	120.1
			
C7—N1—N2—C9	159.24 (10)	C11—C12—C13—C14	−0.57 (17)
C7—N1—N2—C8	−25.33 (16)	C12—C13—C14—C15	−1.30 (16)
C8—N3—N4—C9	0.16 (12)	C12—C13—C14—C17	179.39 (10)
C22—N3—N4—C9	171.99 (10)	C13—C14—C15—C16	1.72 (16)
C6—C1—C2—C3	0.36 (17)	C17—C14—C15—C16	−178.96 (10)
C1—C2—C3—F1	−178.37 (10)	C14—C15—C16—C11	−0.26 (17)
C1—C2—C3—C4	0.72 (18)	+C12—C11—C16—C15	−1.62 (16)
F1—C3—C4—C5	178.21 (10)	C10—C11—C16—C15	172.38 (10)
C2—C3—C4—C5	−0.87 (18)	C13—C14—C17—C18	91.25 (13)
C3—C4—C5—C6	−0.04 (16)	C15—C14—C17—C18	−88.04 (13)
C4—C5—C6—C1	1.07 (16)	C14—C17—C18—C20	−67.35 (12)
C4—C5—C6—C7	−175.07 (10)	C14—C17—C18—C19	169.38 (10)
C2—C1—C6—C5	−1.22 (16)	C29—N5—C22—N3	−51.66 (14)
C2—C1—C6—C7	174.75 (11)	C23—N5—C22—N3	115.30 (11)
N2—N1—C7—C6	−177.20 (9)	C8—N3—C22—N5	129.58 (11)
C5—C6—C7—N1	178.88 (10)	N4—N3—C22—N5	−41.19 (14)
C1—C6—C7—N1	2.87 (16)	C29—N5—C23—C24	113.07 (12)
N4—N3—C8—N2	0.73 (12)	C22—N5—C23—C24	−53.97 (15)
C22—N3—C8—N2	−170.53 (10)	C29—N5—C23—C28	−66.63 (14)
N4—N3—C8—S1	−176.59 (8)	C22—N5—C23—C28	126.33 (11)
C22—N3—C8—S1	12.14 (16)	C28—C23—C24—C25	−0.94 (17)
C9—N2—C8—N3	−1.31 (11)	N5—C23—C24—C25	179.36 (10)
N1—N2—C8—N3	−177.11 (10)	C23—C24—C25—C26	1.27 (18)
C9—N2—C8—S1	175.90 (9)	C24—C25—C26—C27	−0.57 (18)
N1—N2—C8—S1	0.10 (18)	C25—C26—C27—C28	−0.47 (17)
N3—N4—C9—N2	−1.03 (12)	C26—C27—C28—C23	0.79 (17)
N3—N4—C9—C10	−178.02 (10)	C24—C23—C28—C27	−0.08 (17)
C8—N2—C9—N4	1.55 (12)	N5—C23—C28—C27	179.62 (10)
N1—N2—C9—N4	177.97 (9)	C23—N5—C29—C30	−18.94 (15)
C8—N2—C9—C10	178.62 (10)	C22—N5—C29—C30	147.93 (11)
N1—N2—C9—C10	−4.95 (15)	C23—N5—C29—C34	162.97 (10)
N4—C9—C10—C11	105.64 (12)	C22—N5—C29—C34	−30.15 (15)
N2—C9—C10—C11	−70.99 (13)	C34—C29—C30—C31	−0.87 (17)
N4—C9—C10—C21	−17.17 (15)	N5—C29—C30—C31	−178.99 (10)
N2—C9—C10—C21	166.20 (10)	C29—C30—C31—C32	0.09 (18)
C9—C10—C11—C16	138.70 (10)	C30—C31—C32—C33	0.22 (18)
C21—C10—C11—C16	−98.30 (11)	C31—C32—C33—C34	0.25 (18)
C9—C10—C11—C12	−47.47 (13)	C32—C33—C34—C29	−1.04 (18)
C21—C10—C11—C12	75.53 (12)	C30—C29—C34—C33	1.33 (17)
C16—C11—C12—C13	2.04 (16)	N5—C29—C34—C33	179.43 (10)
C10—C11—C12—C13	−171.87 (10)		

### Hydrogen-bond geometry (Å, °)

**Table:**

*D*—H···*A*	*D*—H	H···*A*	*D*···*A*	*D*—H···*A*
C7—H7A···S1	0.93	2.51	3.1983 (11)	131
C4—H4A···Cg1^i^	0.93	2.65	3.5688 (13)	169
C20—H20C···Cg2^ii^	0.96	2.99	3.9291 (12)	166

Symmetry codes: (i) −*x*+1, −*y*+1, −*z*+1; (ii) −*x*+1, *y*+1/2, −*z*+3/2.
